# Supplementary material for: Characterization of Light-Enhanced Respiration in Cyanobacteria
Source: Int J Mol Sci. 2020 Dec 31;22(1):342. doi: 10.3390/ijms22010342 (PMC7796093; doi:10.3390/ijms22010342)
Supplement: Supplementary file 1 [file ijms-22-00342-s001.zip › SupplementalFigure_LEDR_20201227.pdf]

Characterization of light-enhanced respiration in cyanobacteria

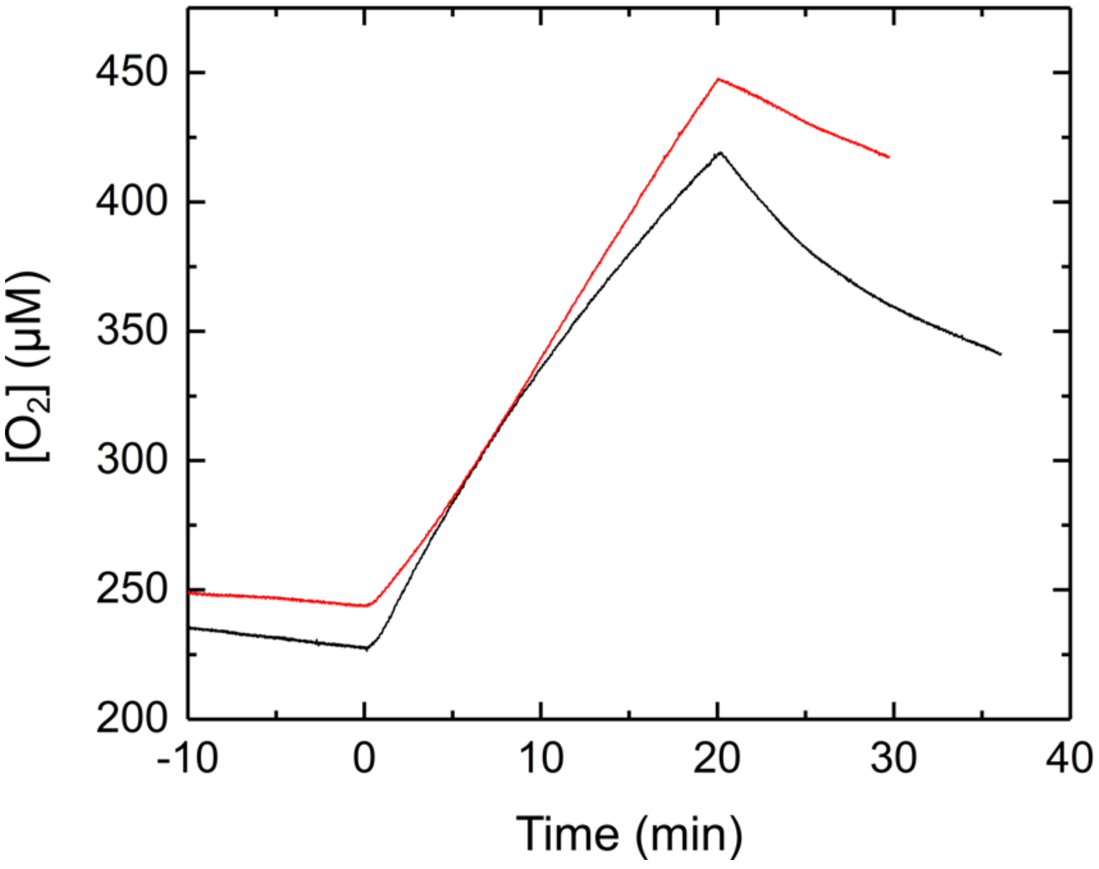

Supplemental Fig. S1. Examples of raw traces of the change in O_2 concentration in *Synechocystis* sp. PCC 6803 wild type (black) and the mutant deficient respiratory terminal oxidases (Δ*cox/cyd/arto*, red). Red actinic light was illuminated for 20 min (190 μmol photons m^{-2} s^{-1}).

Characterization of light-enhanced respiration in cyanobacteria

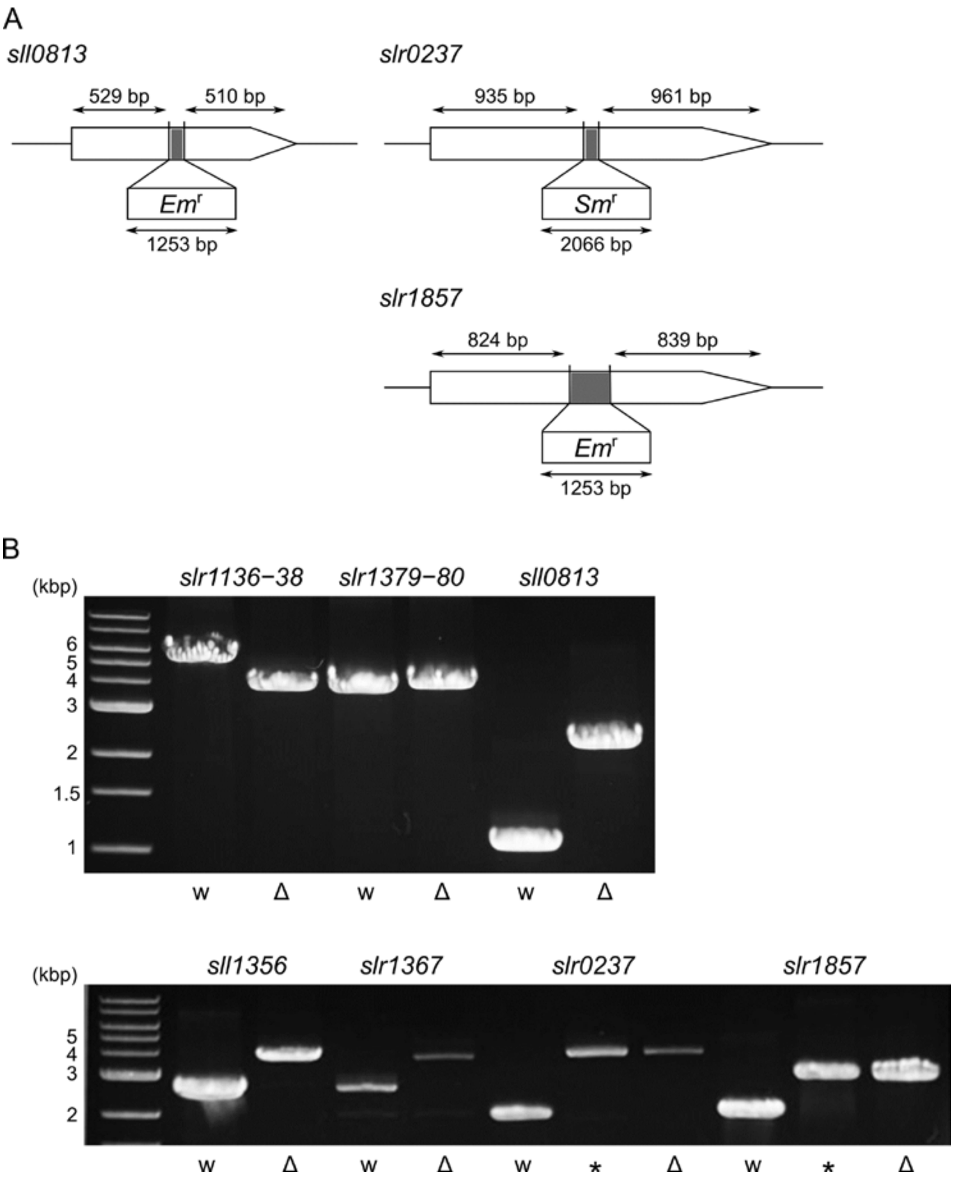

Supplemental Fig. S2. Insertional inactivation of the genes for alternative respiratory terminal oxidase (*arto*) and glycogen deblanching enzymes (*glgX1/2*) in *Synechocystis* sp. PCC 6803. (A) Physical maps of the insertion sites of antibiotic resistance cassettes in each mutant construct. The arrowheads indicate the locations of PCR primers. The lengths of the amplified DNA fragments are shown. The full lengths of *arto* (*sll0813*), *glgX1* (*slr0237*), and *glgX2* (*slr1857*) are 903, 2241 and 2124 bp respectively. The shaded boxes indicate the removed genome DNA regions (*arto*, 34 bp; *glgX1*, 89 bp; *glgX2*, 328 bp). (B) DNA fragments amplified by PCR showing complete segregation of the inactivated genes. Notes: w, wild type; Δ, the mutants used in this study (Δ*cox/cyd/arto* and Δ*glgP/glgX*); *, the double mutant Δ*glgX*. The mutants deficient in *cox* (*slr1136−slr1138*), *cyd* (*slr1379−slr1380*), *glgP1* (*sll1356*), and *glgP2* (*slr1367*) had already been constructed in our previous studies (Shimakawa et al. 2014; Shimakawa and Miyake 2018).

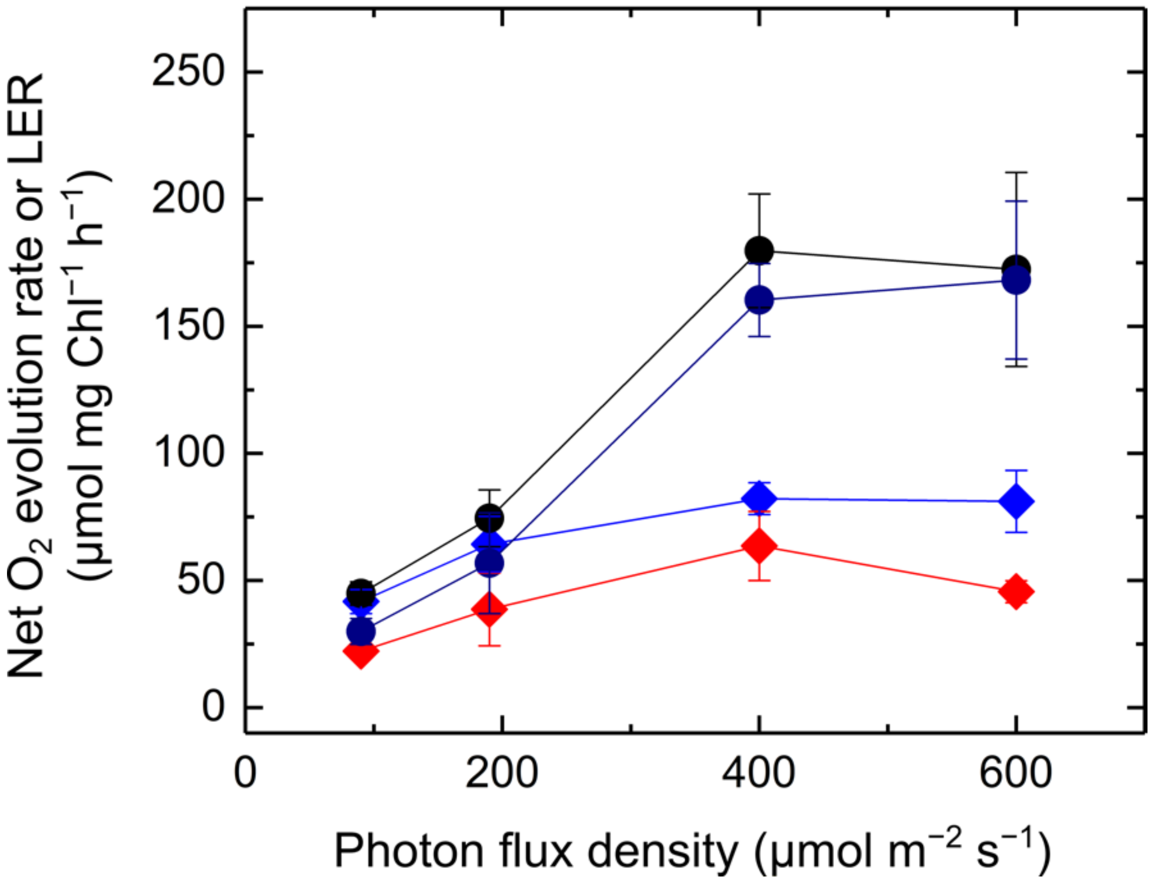

Supplemental Fig. S3. Net O_2 evolution rate (circles) and light-enhanced respiration (LER, diamonds) in the 10 min-illumination with actinic light at different light intensities in *Synechocystis* sp. PCC 6803 adapted to the darkness for 24 h (10 μg Chl mL^{-1}). Experiments were performed in the absence (black, red) and presence (blue, navy) of exogenously added glucose (5 mM). Data are shown as the mean with the standard deviation at 90 (n = 4), 190 (n = 7), 400 (n = 3), and 600 (n = 4) μmol photons m^{-2} s^{-1} (biological replicates).
