# Supplementary material for: Characterization of Light-Enhanced Respiration in Cyanobacteria
Source: Int J Mol Sci. 2020 Dec 31;22(1):342. doi: 10.3390/ijms22010342 (PMC7796093; doi:10.3390/ijms22010342)
Supplement: Supplementary file 1 [file ijms-22-00342-s001.zip › SupplementalTable_LEDR_20201213.pdf]

Characterization of light-enhanced respiration in cyanobacteria

Ginga Shimakawa, Ayaka Kohara, and Chikahiro Miyake

Supplemental Table S1. Primers used in this study

Name	Sequences (5'−3')
sll0813 p1	CTATCTCAAATTCAGCACCGATCAC
sll0813 p2	TTAAAATTGTCCAGACGTAAACGGG
sll0813 p3	TTCCTGTCTTTTTGATGTCGGAAAG
sll0813 p4	TTAATTCGCTGGCCAAAGATAAAGC
slr0237 p1	ATGGGGTTAATTTCTGCTTGTTT
slr0237 p2	CATTTCCGATACCCAGTAGCGCAG
slr0237 p3	CAACATCATCTGGGCGATCGAATC
slr0237 p4	TTCCCAATAGGCATTAAAGATCA
slr1857 p1	GGGGGAGTTAATTTTTCCATTTA
slr1857 p2	GTAATACAAACGGGAGTGAGCATGTAATAGGTTTTGTTAT
slr1857 p3	TTTTTCGTGTGCCTAGATACGGTGCGAAAATTTATTAAAG
slr1857 p4	AGTAGGGATGATTGGGAAGAGAG
Em^r p1	TCCCGTTTGTATTACTTGATCCTT
Em^r p2	TAGGCACACGAAAAACAAGTTAAG
